# Effect of pretransplant spleen volume on the prognosis of acute myeloid leukemia treated with allogeneic hematopoietic stem cell transplantation

**DOI:** 10.3389/fimmu.2025.1675328

**Published:** 2026-02-17

**Authors:** Weiwei Liu, Bingjie Liu, Lihou Pang, Lixia Zhou, Fuxu Wang, Xuejun Zhang, Ying Wang

**Affiliations:** 1 Department of Hematology and Hematology Key Institute, The Second Hospital of Hebei Medical University, Shijiazhuang, Hebei, China; 2 Department of Function, the Second Hospital of Hebei Medical University, Shijiazhuang, Hebei, China; 3 Department of Imaging, the Second Hospital of Hebei Medical University, Shijiazhuang, Hebei, China

**Keywords:** acute myeloid leukemia, hematopoietic stem cell transplantation, non-relapse mortality, prognosis, spleen volume, splenomegaly

## Abstract

**Objective:**

This study aimed to evaluate the influence of pre-transplant spleen volume on the prognosis of patients with acute myeloid leukemia (AML) undergoing allogeneic hematopoietic stem cell transplantation (allo-HSCT).

**Methods:**

We evaluated 58 patients diagnosed with AML and who have undergone first allogeneic stem cell transplant at the Department of Hematology, Second Hospital of Hebei Medical University from 2017 to 2022. All patients were consecutively evaluated. Patients with AML evolving from myeloproliferative neoplasms (MPN) or secondary AML were excluded. Only *de novo* AML patients were included. The median follow-up time was 24 months, and the patients were categorized into non-enlarged spleen volume (NLSV) and large spleen volume (LSV) groups based on the spleen volume ranges of 120 normal individuals. A retrospective analysis was performed to evaluate the impact of spleen volume on post-allo-HSCT outcomes, including the incidence of infection, graft-versus-host disease (GVHD), relapse, overall survival (OS), and non-relapse mortality (NRM). Log-rank test was employed to compare survival curves, and a multivariable Cox regression model was utilized to assess spleen volume as a prognostic factor for long-term survival.

**Results:**

According to the survival curve, the LSV group exhibited lower OS compared to the NLSV group (*P*=0.034), along with higher cumulative NRM (*P*=0.049) and lower relapse-free survival (*P*=0.023). No significant differences were observed in disease relapse, granulocyte engraftment, CMV infection, or GVHD incidence. The multivariable regression model confirmed spleen volume as a significant risk factor for OS, with cytomegalovirus (CMV) infection also influencing OS and NRM.

**Conclusion:**

Pre-transplant splenomegaly is independently associated with poor prognosis in AML patients.

## Introduction

Acute myeloid leukemia (AML) is the most prevalent form of leukemia in adults and represents one of the most significant malignancies of the hematologic system (1). The 5-year survival rate for patients diagnosed with early-stage AML is approximately 30%, with notable variations across different age groups; for instance, the survival rate is less than 10% for patients over 60 years of age (2, 3). The prognosis for AML has improved due to the introduction of novel drugs, targeted therapies, and modifications in treatment protocols. A study examining overall survival (OS) rates in AML over the past 15 years (4) indicates that the 1-, 3-, and 5-year OS rates for patients diagnosed between 2011 and 2019 were 48%, 31%, and 28%, respectively, compared to 42%, 25%, and 22% for those diagnosed from 2004 to 2010. Meanwhile, core binding factor AML has shown improved outcomes with high-dose cytarabine, and certain subtypes of FLT3-ITD mutated AML have responded well to specific targeted therapies, significantly enhancing the prognosis for these subtypes (5–9). Despite these advancements, the overall OS for AML patients remain low at only 25.000% after 5 years, underscoring the ongoing necessity for new drug development and hematopoietic cell transplantation (HCT) (4). Allogeneic hematopoietic stem cell transplantation can enhance survival in AML patients with poor prognosis through the graft-versus-leukemia (GVL) effect; however, it may also increase non-relapse mortality (NRM) due to severe treatment-related complications. Therefore, identifying risk factors for NRM remains critically important (10–15). In patients with myelofibrosis (MF) undergoing allogeneic hematopoietic stem cell transplantation (HSCT), splenomegaly has been demonstrated to adversely impact transplant outcomes, leading to delayed engraftment or failure, increased NRM, and reduced overall survival (16–18).

The relationship between spleen volume and outcomes following hematopoietic stem cell transplantation (HSCT) in patients with acute myeloid leukemia (AML) has garnered increasing attention in recent literature. Several studies highlight the biological and clinical significance of spleen size in the context of AML and HSCT outcomes.

Biological insights into the role of spleen volume are also emerging from cellular and molecular studies. Single-cell RNA sequencing has provided evidence that spleen volume is independently associated with HSCT outcomes in AML, indicating that spleen size may reflect underlying biological patterns influencing transplant success (19). Additionally, immune dysregulation characterized by myeloid-derived suppressor cell (MDSC) expansion in the spleen has been observed in AML, suggesting that spleen enlargement may be linked to immune alterations that impact disease progression and treatment response (20). The pathophysiological mechanisms underlying spleen enlargement in AML involve ineffective hematopoiesis and extramedullary hematopoiesis, which are characteristic features of disease progression and marrow failure (21). In AML evolving from myeloproliferative neoplasms, spleen size has been associated with disease severity and transplant outcomes, emphasizing the biological relevance of spleen volume in disease management (22). Moreover, therapeutic interventions such as azacitidine and MEK inhibitors have demonstrated potential in reducing spleen size, which could translate into improved transplant outcomes (23). In the context of myelofibrosis, the timing of HSCT relative to spleen size is critical. Studies suggest that performing HSCT at the point of optimal spleen response to targeted therapies enhances survival, highlighting the importance of spleen volume as a modifiable factor influencing transplant success (24, 25). Conversely, large spleen size or splenectomy has been associated with increased risks, including poor graft function and survival, underscoring the need for careful pretransplant assessment (22). Overall, the literature indicates that spleen volume is not merely a clinical marker but also reflects underlying biological processes that influence HSCT outcomes in AML. The integration of spleen size assessment into pretransplant evaluation protocols could improve risk stratification and guide therapeutic decision-making, ultimately enhancing patient prognosis (19, 26).

The relationship between pre-transplant spleen size and prognosis in patients with acute myeloid leukemia (AML) remains inadequately understood. The effect of splenomegaly on prognosis for AML patients undergoing hematopoietic stem cell transplantation (HSCT) is still a matter of debate. In this study, we retrospectively examined the associations between pre-transplant spleen size and various post-transplant outcomes, including overall survival (OS), cumulative incidence of relapse (CIR), non-relapse mortality (NRM), and relapse-free survival (RFS).

## Materials and methods

### Study populations

The study evaluated 100 AML patients undergoing their first HSCT at the Second Hospital of Hebei Medical University between January 1, 2017 and January 1, 2022. All patients were consecutively evaluated. Patients with AML evolving from myeloproliferative neoplasms (MPN) or secondary AML were excluded. Only *de novo* AML patients were included. A total of 42 patients were excluded due to missing pre-transplant abdominal CT scans. The final cohort included 58 patients: 27 men (46.552%) and 31 women (53.448%), with a median age of 37 years (range: 15–59 years). No patient had evidence of splenic vein thrombosis on imaging. Pre-transplant conditioning and GVHD prophylaxis followed the Beijing Protocol (27). Patients without extramedullary involvement received modified Bu/Cy (busulfan 3.2 mg/kg/day on days –8 to –6; cyclophosphamide (Cy) 1.8 g/m²/day on days –5 to –4). Those with extramedullary involvement received TBI-Cy/fludarabine (Flu) (total body irradiation 4.0 Gy/day on days –7 to –6; Cy 1.8 g/m²/day on days –5 to –4 or Flu 30 mg/m²/day on days –5 to –2). Doses were adjusted based on individual patient conditions. A total of 24 patients received HLA-identical donor transplants and 34 received haplo-identical donor transplants. On day 0, either PBSC or BMSC was infused. The target dose for haploid transplantation is ≥5.0 × 10^6^/kg of CD34^+^ cells, while the target dose for HLA-matched transplantation is ≥2.0 × 10^6^/kg. The actual infusion dose is adjusted based on the donor’s mobilization status and the patient’s body weight. The GVHD prophylaxis regimens were as follows: for HLA-identical HSCT, short-course methotrexate (MTX) + cyclosporine A (CSA) + mycophenolate mofetil (MMF) were used. If the donor was a woman over 40 years old, ATG (2.5 mg/kg) was added on days –3 and –2 (28). For haplo-identical HSCT, the regimen included short-course MTX + CSA + MMF + ATG (2.5 mg/kg on days –5 to –2) (27). Pre-transplant disease status was classified as complete remission (CR) or partial remission (PR) with minimal residual disease (MRD) in AML patients detected by flow cytometry as below 2.5% (29). Acute and chronic GVHD were diagnosed and treated according to standard protocols (15–17). Engraftment was defined as follows: neutrophil engraftment: ≥0.5 × 10^9^/L for three consecutive days; platelet engraftment: ≥ 20 × 10^9^/L for seven consecutive days without transfusion. Relapse: Blasts reappear in blood, bone marrow (>5%), or extramedullary sites after CR; PR was defined as a reduction in the proportion of myeloid cells to 5%–25% and a decrease of at least 50% from the baseline level. MRD was monitored at +1, +1.5, +2, +3, +4, +6, +9, +12, +18, +24, +36, and +48 months post-transplantation. MRD management at +1.5 months: Low- to intermediate-risk patients with positive MRD received DLI; low- to intermediate-risk patients with negative MRD and no GVHD received DLI; intermediate- and high-risk patients with negative MRD and GVHD received DLI at 1 × 10^7^ CD3+ cells/kg. If MRD was negative but GVHD was present, the CsA dose was reduced. Patients with positive MRD and no GVHD received DLI + AZA (32–50 mg/kg/day for 5 days) (30, 31).

### Measurement of spleen volume

**Figure 1:**
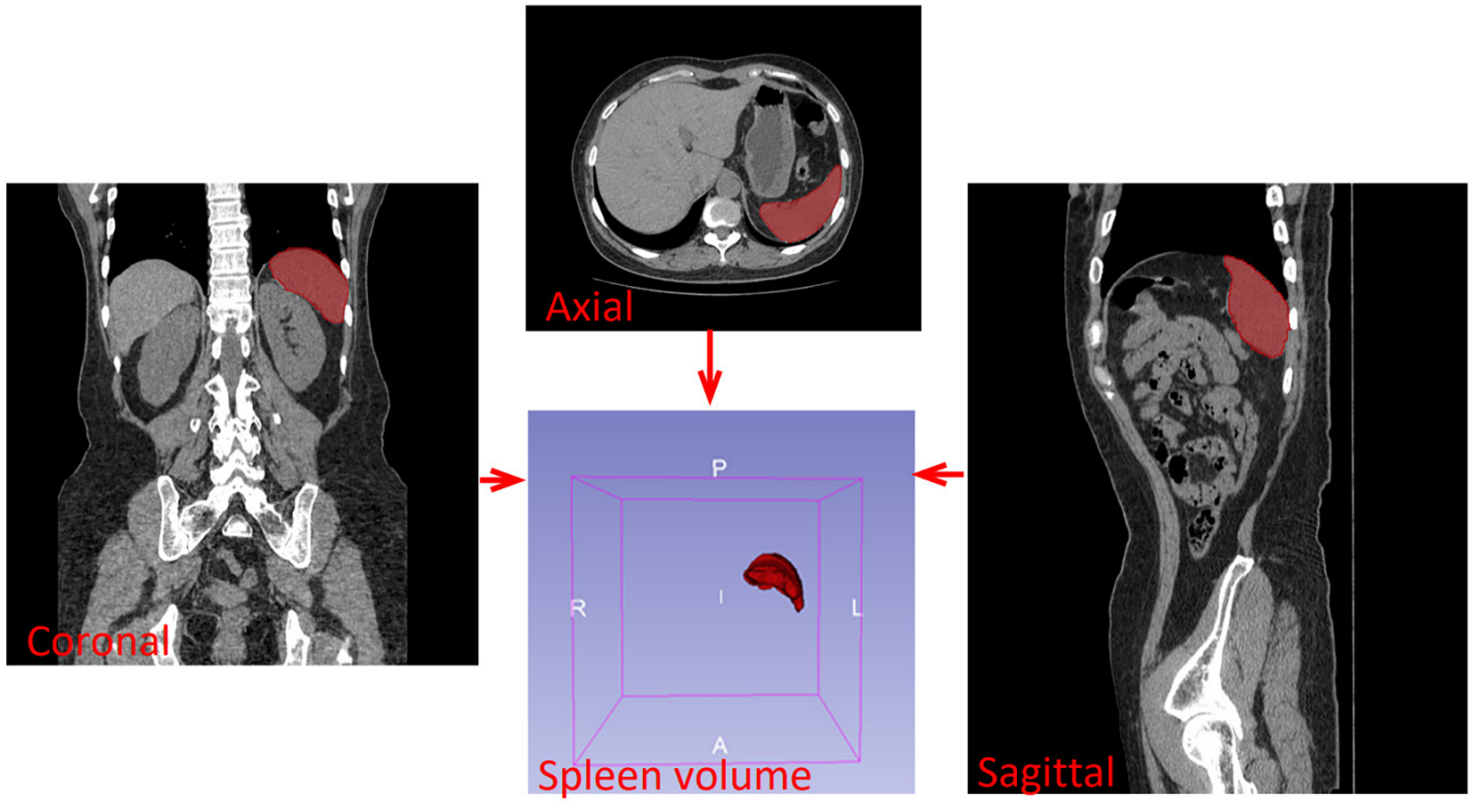
Measurement of spleen volume.

Spleen volume measurements were obtained from abdominal CT scans performed within 30 days prior to transplantation. CT scanning was conducted using a dual-source 64-row spiral CT (Philips CT, Second Hospital of Hebei Medical University), with the dataset reconstructed to a slice thickness of 1.250 mm. The contour of the spleen was delineated based on the density differences between the spleen and the surrounding tissues. The examiner was blinded to the patient’s disease and clinical status. Spleen volume was calculated and analyzed using the label map statistics plug-in in 3D Slicer 5.4.0 software. For optimal measurement, three planes of scanned images were obtained: coronal, sagittal, and axial (**Figure 1**).

### Statistical analysis

A total of 120 normal controls were paired with the case group at a ratio of 1:2 in terms of height, weight, and gender, thereby establishing the normal value range of CT spleen volume for the normal control group. Healthy controls were selected from patients without hematological disorders who underwent upper abdominal CT and ultrasound imaging during routine clinical evaluation. All imaging data were anonymized in compliance with ethics committee requirements. Inclusion criteria included the absence of hematological disease and ultrasonographically confirmed normal spleen size, as defined by established reference values (32). Controls were matched to the case group based on key demographic and anthropometric characteristics, including sex, height, and body weight. All of the controls had no history of diseases that affected the size of the spleen. This study has been approved by the Research Ethics Committee of the Second Hospital of Hebei Medical University (approval number: 2025-R032), and it has followed relevant laws, regulations, and ethical principles. The spleen volume was measured using the aforementioned method. The 95.000% confidence interval for normal spleen volume was determined through a one-sample *t*-test, yielding a range of 193.690–223.640 cm³. The cohort was divided into the non-enlarged spleen volume (NLSV) group and the large spleen volume (LSV) group based on the upper limit of normal spleen volume (223.640 cm³). Categorical variables were analyzed using chi-square test and Mann–Whitney *U*-test. Overall survival (OS), non-relapse mortality (NRM), and cumulative incidence of relapse (CIR) were estimated using the Kaplan–Meier method, and the probability of survival within 2 years was calculated. Survival curves were compared with the log-rank test. A multivariable Cox regression model was developed to assess the potential risk of spleen volume as a prognostic factor for OS, CIR, and NRM. The adjusted variables included age, gender, acute myeloid leukemia (AML) subtype, HLA matching, donor type, disease remission status, and cytomegalovirus (CMV) infection. Statistical analyses were conducted using SPSS version 21.0 and GraphPad Prism version 8, with a significance threshold set at *P <*0.050.

## Results

A total of 58 patients were included in this analysis, with an average spleen volume of 305.360 cm³ (range: 84.630–1148.650 cm³). The cohort was stratified into two groups based on the upper limit of normal spleen volume: the normal spleen volume (NLSV) group (spleen volume ≤223.640 cm³) and the large spleen volume (LSV) group (spleen volume >223.640 cm³). The basic characteristics of the patients, categorized by spleen volume, are presented in **Table 1**.

**Table 1:** Basic characteristics of the patients.

Variable	NLSV	LSV	Total	P-value
Quantity	21	37	58	
Age (years)				
Median (Range)	37 (16-53)	31 (15-59)	37 (15-59)	
Gender (%)				0.040
Male	6 (28.600)	21 (56.800)	27 (46.552)	
Female	15 (71.400)	16 (43.200)	31 (53.448)	
ELN 2022 risk (%)				0.099
Favorable	0 (0.000)	0 (0.000)	0 (0.000)	
Intermediate	15 (71.429)	17 (45.946)	32 (55.172)	
Adverse	6 (28.571)	20 (54.054)	26 (44.823)	
Donor HLA Match (%)				0.704
Fully Matched	8 (38.095)	16 (43.243)	24 (41.379)	
Half Matched	13 (61.905)	21 (56.757)	34 (58.620)	
Pre-transplant Disease Status (%)				0.388
CR	10 (47.619)	22 (59.460)	32 (55.172)	
PR/NR	11 (52.381)	15 (40.541)	26 (44.828)	
CMV Infection (%)				0.354
Yes	10 (47.619)	13 (35.135)	23 (39.655)	
No	11 (52.381)	24 (64.865)	35 (60.345)	
GVHD (%)				0.262
Present	7 (33.300)	18 (48.649)	25 (43.104)	
Absent	14 (66.667)	19 (51.351)	33 (56.897)	
Relapse (%)				0.245
Yes	2 (9.524)	8 (21.622)	10 (17.241)	
No	19 (90.476)	29 (78.378)	48 (82.759)	

Non-enlarged spleen volume (NLSV) was defined as spleen volume less than or equal to 223.64 cm^3^, and large spleen volume (LSV) was defined as spleen volume greater than 223.64 cm^3^. Medians and ranges were given for continuous variables and percentages for categorial variables.

**Figure 2:**
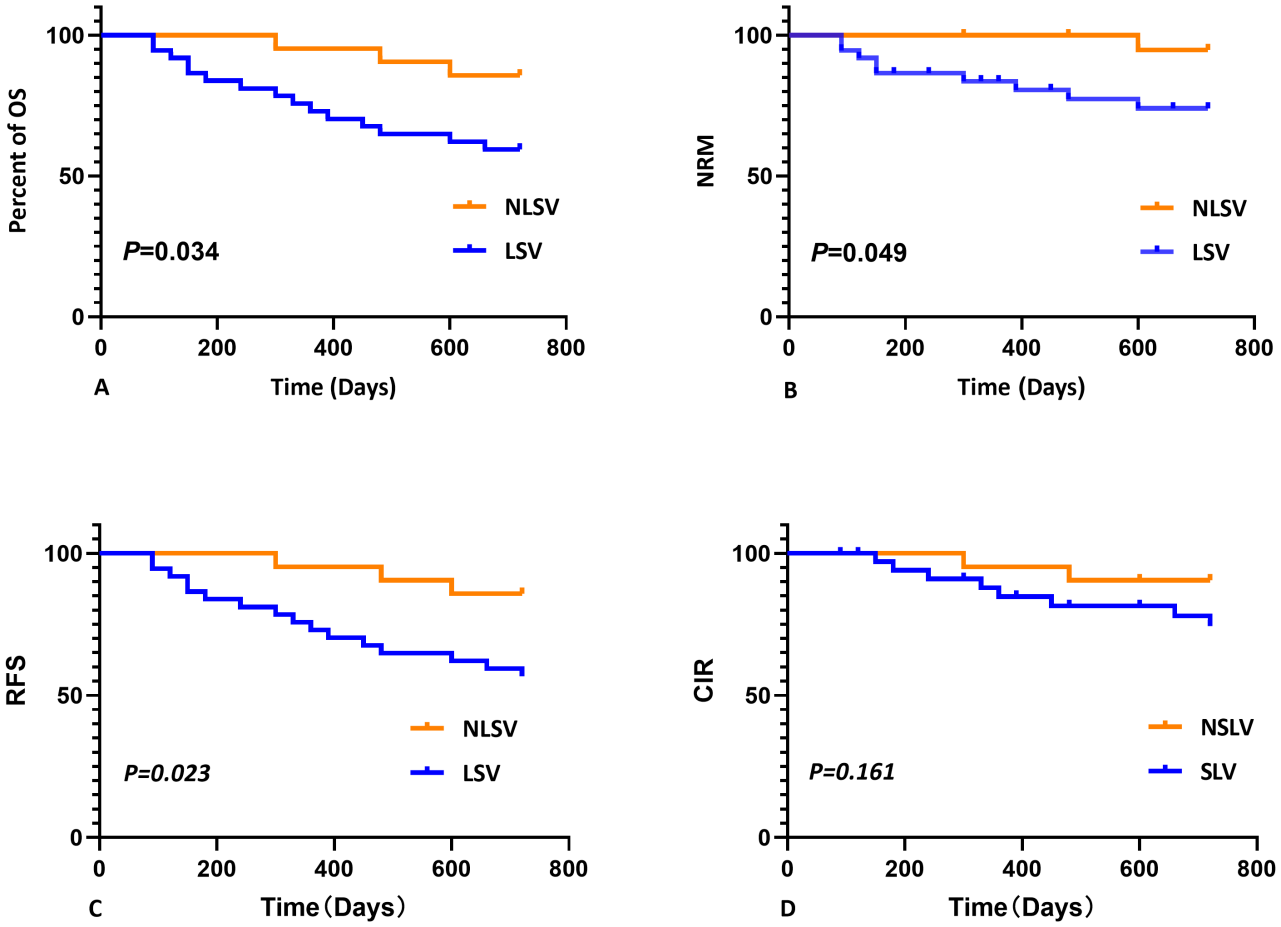
Kaplan-Meier analysis of overall survival (OS), non-relapse mortality (NRM), relapse-free survival (RFS), and cumulative incidence of relapse (CIR) stratified by spleen size. **(A)** Kaplan–Meier analysis of OS stratified by spleen size. The 2-year OS was shorter for LSV (59.459%) compared with NLSV (85.714%), *P*=0.034. **(B)** Kaplan–Meier analysis of NRM stratified by spleen size. The 2-year NRM was longer for LSV (25.933%) compared with NLSV (5.263%), *P*=0.049. **(C)** Kaplan–Meier analysis of RFS stratified by spleen size. The 2-year RFS was shorter for LSV (56.757%) compared with NLSV (85.741%), *P*=0.023. **(D)** Kaplan–Meier analysis of RCIR stratified by spleen size. The 2-year CIR was shorter for LSV (25.624%) compared with NLSV (9.524%), *P*=0.161.

**Figure 3:**
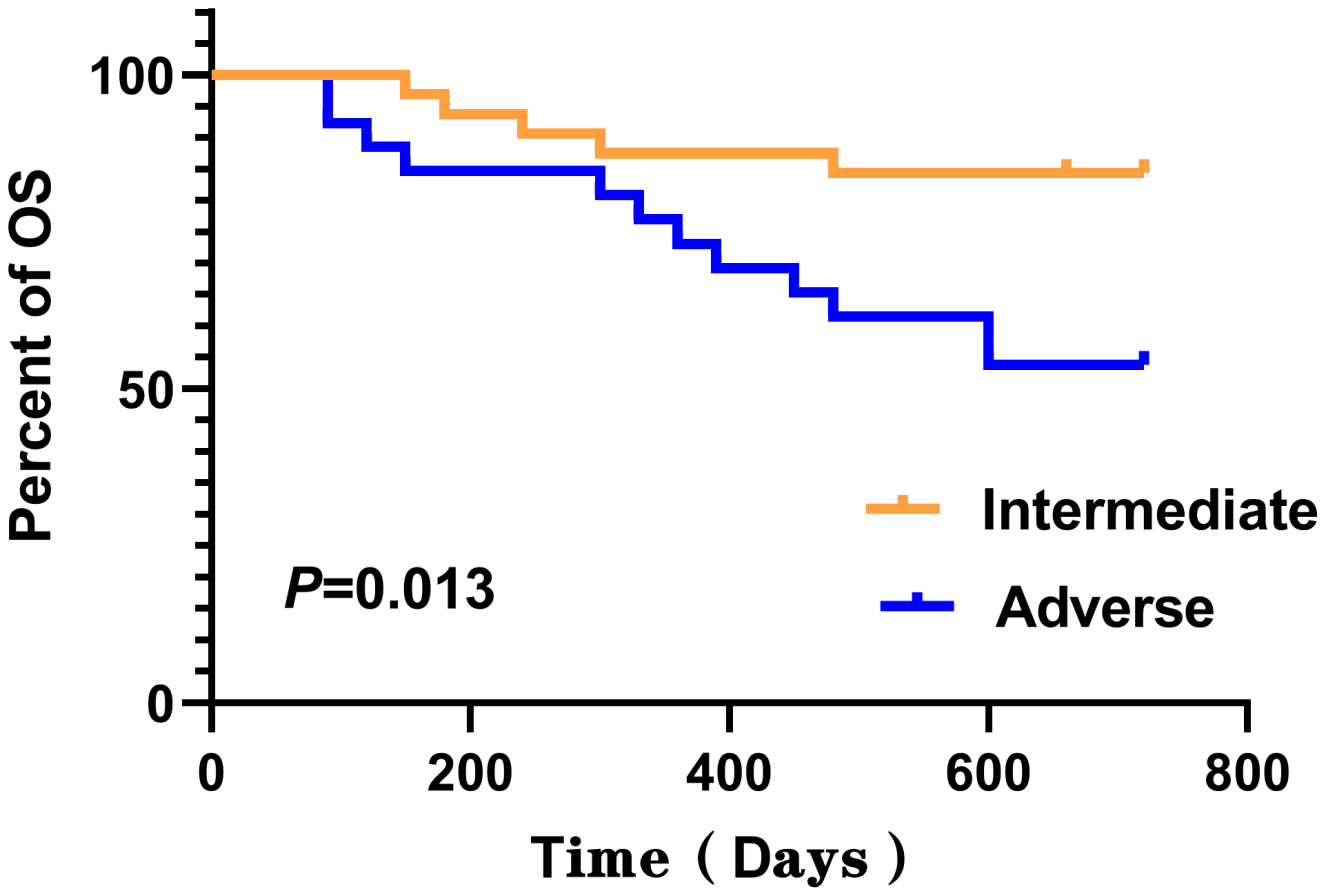
Kaplan–Meier analysis of overall survival (OS) stratified by ELN 2022 risk stratification. The 2-year OS was shorter for adverse (53.846%) compared with intermediate (84.375%), *P*=0.013.

**Figure 4:**
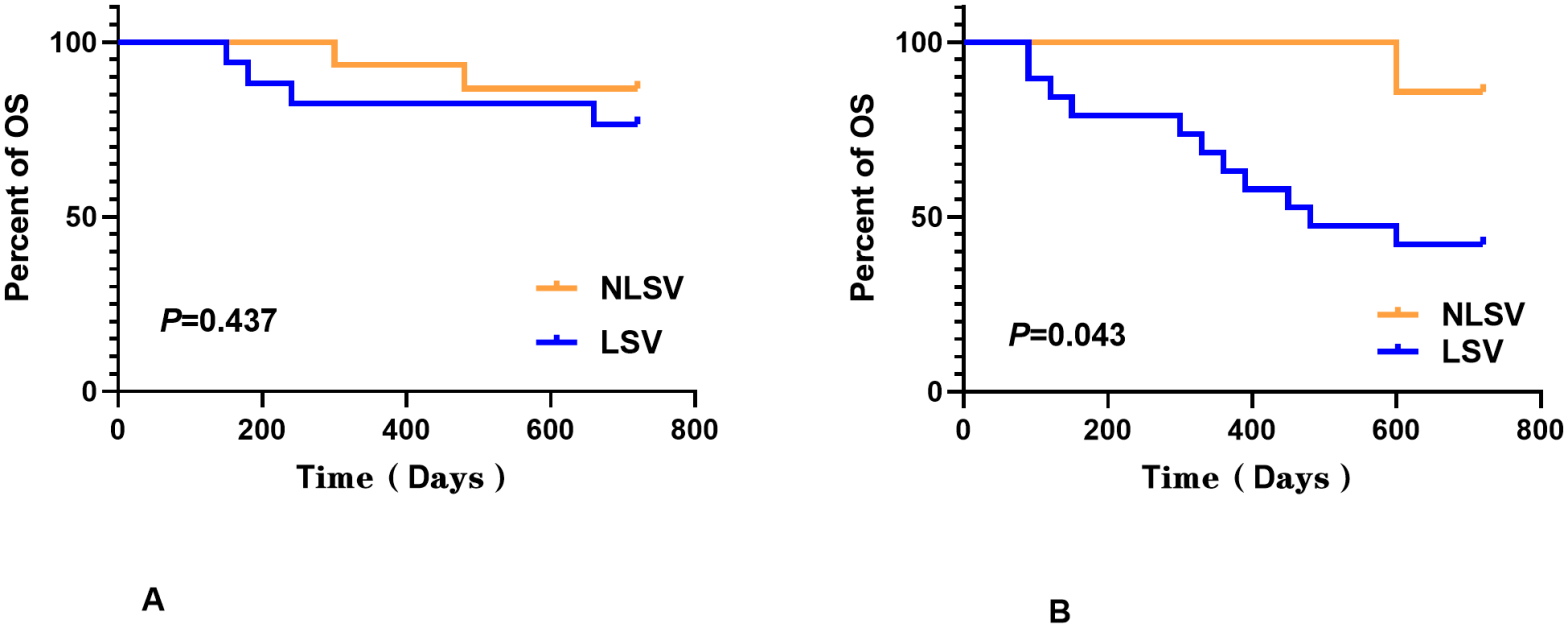
ELN grading (classified as NLSV versus LSV) on patient survival outcomes. In the intermediate-risk subgroup **(A)**, no statistically significant difference in overall survival was observed between the NLSV and LSV groups (*P*=0.437), while in the high-risk group **(B)**, the LSV patients showed significantly lower overall survival rates than the NLSV patients (*P*=0.043).

No statistically significant differences were detected between the two groups for any of the analyzed features except for gender, as determined by Mann–Whitney *U*-test (*P* > 0.050). Log-rank test was employed to assess the differences in survival curves between the two groups. Study endpoints included NRM, TRM, aGVHD, OS, and DFS. Terms are defined as follows: NRM, death from any cause other than relapse; TRM, death due to transplant-related complications; OS, time from transplantation to death from any cause; DFS, survival with continuous CR. The results indicated that the overall survival (OS) of the LSV cohort was significantly lower than that of the NLSV cohort (59.459% vs. 85.714% at 2 years; *P*=0.034) (**Figure 2A**). In addition, the non-relapse mortality (NRM) in the LSV group was higher than that in the NLSV group (25.933% vs. 5.263%; *P*=0.049; **Figure 2B**), and the relapse-free survival (RFS) was significantly lower in the LSV cohort compared with the NLSV group (56.757% vs. 85.741%; *P*=0.023) (**Figure 2C**). Although the LSV group exhibited a higher cumulative incidence of recurrence (CIR) compared to the NLSV group (25.624% vs. 9.524%; *P*=0.161), this difference was not significant (**Figure 2D**). The overall survival analysis of the ELN 2022 risk stratification system (intermediate vs. adverse) (**Figure 3**) revealed that the overall survival rate in the adverse group was significantly lower than that in the intermediate group (*P*=0.013), further validating the effectiveness of the ELN grading system in prognostic stratification. To further evaluate the impact of ELN grading (classified as NLSV versus LSV) on patient survival outcomes, we performed stratified survival analyses for intermediate-risk (intermediate) and high-risk (adverse) groups. In the intermediate-risk subgroup (**Figure 4A**), no statistically significant difference in overall survival was observed between NLSV and LSV groups (*P*=0.437), suggesting that splenomegaly size may not be an independent prognostic factor in this population. Conversely, in the high-risk group (**Figure 4B**), LSV patients showed significantly lower overall survival rates than NLSV patients (*P*=0.043), indicating that splenomegaly serves as a critical prognostic indicator for patients with an elevated risk of adverse outcomes. Spleen volume holds a significant prognostic value in high-risk ELN patients. In future clinical risk assessments and treatment strategy development, the combined use of spleen volume and ELN grading can be comprehensively considered. The results of the survival analysis suggest that an increased spleen volume is associated with reduced overall survival (OS) and increased non-relapse mortality (NRM) in patients with acute myeloid leukemia (AML) who have undergone transplantation, leading to a poorer prognosis. No significant differences were observed in disease relapse or granulocyte engraftment. Furthermore, given that the spleen volume of men with the same height and weight is marginally larger than that of women, to mitigate this bias, we selected the normal control group at a 1:2 ratio based on the same height, weight, and gender. As a result, a spleen larger than the upper limit of the normal control was defined as splenomegaly, which made it more definite that the spleen in the LSV group was larger than that of normal individuals. On the other hand, when chemotherapy was the treatment background for AML, the OS of male patients was slightly inferior to that of female patients (33), but when transplantation was the treatment background, this difference became non-significant (34). Hence, although the proportion of male patients was significantly higher in the LSV group than that in the NLSV group, the influence of gender bias on the prognosis of transplantation treatment can be disregarded.

The multivariate Cox proportional hazards regression analysis (**Table 2**) indicated that spleen volume remained independently associated with 2-year overall survival (*P*=0.012) after adjusting for age, sex, ELN risk stratification, donor HLA matching, pre-transplant disease remission status, and cytomegalovirus (CMV) infection. The hazard ratio (HR) for overall survival in the low spleen volume (LSV) group was 6.099 (95% confidence interval [CI], 1.494–24.904) when compared to the non-low spleen volume (NLSV) group. In addition to spleen volume, CMV infection is another co-factor that affects overall survival (OS) and non-relapse mortality (NRM). Relative to the NLSV group, the adjusted HR for NRM in the LSV group was 10.108 (95% CI, 0.951–107.453). However, despite the higher HR for NRM, no significant correlation was found (*P*=0.055). In conjunction with the preceding analysis of survival curves, it is still posited in clinical practice that spleen volume may be a significant influencing factor on NRM, potentially due to the limited number of cases and the short follow-up duration. Furthermore, the multivariate regression analysis revealed no significant correlation between spleen volume and cumulative incidence of relapse (CIR) (*P*=0.073; **Table 2**).

**Table 2:** Multivariate analysis of overall survival, non-relapse mortality, and cumulative relapse rate.

Category	OS		NRM		CIR	
	HR (95%CI)	P	HR (95%CI)	P	HR (95%CI)	P-Value
Age	1.037 (0.989,1.086)	0.131	1.022 (0.962,1.086)	0.476	1.049 (0.973,1.130)	0.212
Gender (Male/Female)	1.284 (0.480,3.437)	0.618	3.666 (0.691,19.440)	0.127	0.416 (0.101,1.708)	0.224
ELN risk (Intermediate/Adverse)	0.2919 (0.110,0.772)	0.013	0.08518 (0.02239, 0.3241)	0.003	1.516 (0.4265,5.387)	0.520
Donor HLA Match (Fully/Half)	0.932 (0.335,2.598)	0893	4.775 (0.507,44.985)	0.172	0.308 (0.069,1.367)	0.121
Pre-transplant Disease Status (PR or NR/CR)	2.524 (0.921,6.915)	0.072	3.008 (0.792,12.419)	0.128	2.809 (0.693,11.375)	0.148
CMV Infection (Yes/No)	2.885 (1.015,8.198)	0.047	9.412 (1.682,52.670)	0.011	0.844 (0.159,4.491)	0.554
Spleen Volume (LSV/NLSV)	6.099 (1.494,24.904)	0.012	10.108 (0.951,107.453)	0.055	4.637 (0.865,24.846)	0.073

To further investigate the potential role of spleen volume as a risk factor for OS and NRM, we excluded the influence of CMV infection on relapse. Patients without CMV infection were categorized based on the presence or absence of disease relapse, while patients without disease recurrence were classified according to the presence or absence of CMV infection (**Table 3**).

**Table 3:** Relationship between spleen volume and infection and recurrence.

Category	LSV	NLSV	*P*-value
Relapse (no CMV infection)			1.000
Yes	6	2	
No	18	9	
CMV Infection (no relapse)			0.380
Yes	11	10	
No	18	9	

Following the more precise categorization of patients (**Table 3**), our findings revealed no significant differences in the rates of relapse and cytomegalovirus (CMV) infection between the two groups (*P*=1.000 and *P*=0.380, respectively). This suggests that the interaction between CMV infection and recurrence is not a significant factor.

## Discussion

Acute myeloid leukemia (AML) is the most prevalent form of acute leukemia among adults, and for the majority of AML patients, allogeneic hematopoietic stem cell transplantation (allo-HSCT) represents the only potentially curative treatment option (35). Consequently, it is essential to identify factors that influence the prognosis of allo-HSCT to enhance long-term survival rates. By monitoring pertinent factors and developing risk stratification, we can establish effective prevention strategies and targeted therapies, thereby improving patient survival and quality of life. A study investigating the impact of splenomegaly on HSCT outcomes in patients with chronic myelomonocytic leukemia (CMML) (36) revealed that patients with splenomegaly experienced a longer median time to neutrophil engraftment compared to those without splenomegaly (*P*=0.021). However, splenomegaly did not appear to affect disease relapse, overall survival (OS), or disease-free survival. Chen Juan et al. (37) found that a CD3+/CD8+ cell ratio <14.825% in haploidentical donor lymphocytes was linked to a higher CMV infection risk within 100 days after HSCT, but not to 1-year relapse. Moreover, evidence shows that factors like HLA typing, conditioning regimen intensity, and use of immunosuppressive agents or ATG affect GVHD incidence and severity (38–40). A study by Lindandan et al. (41) found that gut microbiota is linked to acute GVHD development. Dysbiosis and altered microbial metabolites are common in acute GVHD and correlate with poor outcomes. Antibiotics before transplantation may change the gut microbiota composition in patients with hematologic cancers. Lee MW et al. (42) found that the pre-transplant use of glycopeptide antibiotics may increase the risk of extensive chronic GVHD (cGVHD). Splenomegaly has been associated with primary implant failure in patients with myelofibrosis, but how splenomegaly affects patients after HSCT for myeloid malignancies is unclear. Our current study found that increased spleen volume was associated with poor prognosis after HSCT in AML patients. At follow-up 2 years post-transplantation, between-group survival analysis showed that OS was 85.714% in the NLSV group and 59.459% in the LSV group (*P*=0.034). Meanwhile, NRM was higher in the LSV patients, whereas CIR did not differ between them. Splenomegaly is linked to implant failure in myelofibrosis, but its effect on HSCT outcomes in myeloid malignancies is unclear. Our study found that a larger spleen volume correlates with worse prognosis after HSCT in AML patients. At 2 years post-transplantation, OS was 85.714% in the NLSV group and 59.459% in the LSV group (*P*=0.034). NRM was higher in the LSV group, but CIR did not differ (*P*=0.161). This suggests that spleen volume does not affect survival through recurrence. How splenomegaly increases NRM remains unknown. Spleen size may also be influenced by age, gender, and BMI. In the study, the proportion of male patients was significantly higher in the LSV group than in the NLSV group. It is known that overall survival (OS) was slightly lower in male patients than in female patients when chemotherapy was used as the treatment background (33), but this difference became insignificant when transplantation was used as the treatment background (34). Thus, in this study, the effect of gender bias on transplantation outcomes was negligible. Meanwhile, after adding donor HLA matching type, remission status before transplantation, and CMV infection to multivariate regression analysis, splenic volume remained independently associated with OS; however, we observed that CMV infection is also an important factor affecting patient prognosis. Therefore, this study supports that splenomegaly is an independent risk factor for OS. In terms of non-relapse mortality (NRM), we did not find a significant difference (*P*=0.055) despite a higher hazard ratio (HR). Combined with survival curve analysis, we believe clinically that splenic volume is a potentially meaningful influencing factor for NRM; this may be related to our small sample size and shorter follow-up time. Further work is needed to verify the influence of splenomegaly on NRM for patients undergoing HSCT. The mechanisms by which splenomegaly affects post-transplant survival are not well understood. Chen et al. (43) reported that splenomegaly was independently linked to primary graft failure (PGF) after allo-HSCT (*P*=0.030) and that PGF was associated with significantly lower overall survival (OS) (*P*=0.001). Pohlmann et al. (26) found that patients with spleen volume >238,000 cm³ had lower 2-year OS (55.7% vs. 66.6%; *P*=0.009) and higher non-relapse mortality (NRM) (28.8% vs. 20.2%; *P*=0.048), However, this study did not use a normal control group matched by height, weight, and age to define the size of the spleen. There were no significant differences in engraftment times or rates of acute or chronic GVHD. Song et al. (44) also found that splenomegaly was independently associated with platelet transfusion refractoriness (*P* < 0.001), which reduced OS. The spleen, as the body’s largest immune organ, may affect patient survival through immune mechanisms involving natural killer (NK) cells—important cells in fighting infections and tumors. A study (45) showed that NK cells make up a large portion of lymphocytes in blood, bone marrow, spleen, and lung, mainly displaying a CD56dim CD16+ phenotype. In contrast, fewer NK cells are found in lymph nodes, tonsils, and intestines, where they mostly show a CD56bri CD16– phenotype. These findings suggest that the spleen is a major site for NK cells, especially the CD56dim CD16+ type. Splenomegaly may trap more NK cells in the spleen, possibly reducing their levels and function in the blood. Delayed recovery of donor NK cells, along with fewer and less active NK cells from an enlarged spleen, could increase the risk of infection and disease relapse after transplantation.

In our AML patient cohort, we observed more deaths from viral infections. Splenomegaly may suppress the immune system, leading to more severe post-transplant infections and worse overall survival (OS). However, this study is a single-center retrospective study with a small sample size, which may introduce selection bias. Additionally, spleen volume measurement may exhibit variability. In the future, we will continue to conduct multicenter, prospective, large-sample studies to further explore its mechanisms, incorporating techniques such as spleen imaging omics and immunomics. Future multicenter prospective studies will help further validate our findings and explore the specific mechanisms by which spleen volume affects HSCT outcomes.

## Conclusions

Splenomegaly is an independent risk factor for poor outcomes in AML patients undergoing allogeneic HSCT. It mainly affects overall survival (OS) and may also increase the risk of non-relapse mortality (NRM), though it does not significantly hinder stem cell engraftment. Studies show that splenomegaly is linked to worse OS in transplant recipients. Possible reasons include delayed stem cell engraftment and changes in natural killer (NK) cell levels and function, which weaken the immune system. These factors lead to longer transfusion needs, higher risks of bleeding and infection, and more relapses—ultimately resulting in lower survival rates. In summary, our findings highlight the importance of spleen size as a key prognostic factor in AML transplantation and offer a new method to assess transplant candidates before the procedure.

## Data Availability

The original contributions presented in the study are included in the article/supplementary material. Further inquiries can be directed to the corresponding author.
