# Correction: Effect of pretransplant spleen volume on the prognosis of acute myeloid leukemia treated with allogeneic hematopoietic stem cell transplantation

**DOI:** 10.3389/fimmu.2026.1889158

**Published:** 2026-07-02

**Authors:** Weiwei Liu, Bingjie Liu, Lihou Pang, Lixia Zhou, Fuxu Wang, Xuejun Zhang, Ying Wang

**Affiliations:** 1 Department of Hematology and Hematology Key Institute, The Second Hospital of Hebei Medical University, Shijiazhuang, Hebei, China; 2 Department of Function, the Second Hospital of Hebei Medical University, Shijiazhuang, Hebei, China; 3 Department of Imaging, the Second Hospital of Hebei Medical University, Shijiazhuang, Hebei, China

**Keywords:** acute myeloid leukemia, hematopoietic stem cell transplantation, non-relapse mortality, prognosis, spleen volume, splenomegaly

There was a mistake in (**Table 1**) as published. (**Table 1**) (“Basic characteristics of the patients”) still reflects the original format which was an old version and the 2022 ELN risk were not included. Therefore, we revised the format. The corrected (**Table 1**) appears below.

**Table 1:** Basic characteristics of the patients.

Variable	NLSV	LSV	Total	P-value
Quantity	21	37	58	
Age (years)				
Median (Range)	37 (16-53)	31 (15-59)	37 (15-59)	
Gender (%)				0.040
Male	6 (28.600)	21 (56.800)	27 (46.552)	
Female	15 (71.400)	16 (43.200)	31 (53.448)	
ELN 2022 risk (%)				0.099
Favorable	0 (0.000)	0 (0.000)	0 (0.000)	
Intermediate	15 (71.429)	17 (45.946)	32 (55.172)	
Adverse	6 (28.571)	20 (54.054)	26 (44.823)	
Donor HLA Match (%)				0.704
Fully Matched	8 (38.095)	16 (43.243)	24 (41.379)	
Half Matched	13 (61.905)	21 (56.757)	34 (58.620)	
Pre-transplant Disease Status (%)				0.388
CR	10 (47.619)	22 (59.460)	32 (55.172)	
PR/NR	11 (52.381)	15 (40.541)	26 (44.828)	
CMV Infection (%)				0.354
Yes	10 (47.619)	13 (35.135)	23 (39.655)	
No	11 (52.381)	24 (64.865)	35 (60.345)	
GVHD (%)				0.262
Present	7 (33.300)	18 (48.649)	25 (43.104)	
Absent	14 (66.667)	19 (51.351)	33 (56.897)	
Relapse (%)				0.245
Yes	2 (9.524)	8 (21.622)	10 (17.241)	
No	19 (90.476)	29 (78.378)	48 (82.759)	

There was a mistake in (**Table 2**) as published. (**Table 2**) (“Multivariate analysis of overall survival, non-relapse mortality, and cumulative relapse rate.” still reflects the original format which was an old version and the 2022 ELN risk were not included. Therefore, we revised the format. The corrected (**Table 2**) appears below.

**Table 2:** Multivariate analysis of overall survival, non-relapse mortality, and cumulative relapse rate.

Category	OS		NRM		CIR	
	HR (95%CI)	P	HR (95%CI)	P	HR (95%CI)	P-Value
Age	1.037 (0.989,1.086)	0.131	1.022 (0.962,1.086)	0.476	1.049 (0.973,1.130)	0.212
Gender (Male/Female)	1.284 (0.480,3.437)	0.618	3.666 (0.691,19.440)	0.127	0.416 (0.101,1.708)	0.224
ELN risk (Intermediate/Adverse)	0.2919 (0.110,0.772)	0.013	0.08518 (0.02239, 0.3241)	0.003	1.516 (0.4265,5.387)	0.520
Donor HLA Match (Fully/Half)	0.932 (0.335,2.598)	0893	4.775 (0.507,44.985)	0.172	0.308 (0.069,1.367)	0.121
Pre-transplant Disease Status (PR or NR/CR)	2.524 (0.921,6.915)	0.072	3.008 (0.792,12.419)	0.128	2.809 (0.693,11.375)	0.148
CMV Infection (Yes/No)	2.885 (1.015,8.198)	0.047	9.412 (1.682,52.670)	0.011	0.844 (0.159,4.491)	0.554
Spleen Volume (LSV/NLSV)	6.099 (1.494,24.904)	0.012	10.108 (0.951,107.453)	0.055	4.637 (0.865,24.846)	0.073

There were mistakes in the caption of **Figure 3, 4** as published. The abbreviation “ENL” was used incorrectly instead of “ELN”. The corrected caption of **Figure 3** appears below.

“Kaplan–Meier analysis of overall survival (OS) stratified by ELN 2022 risk stratification. The 2-year OS was shorter for adverse (53.846%) compared with intermediate (84.375%), P = 0.013.

The corrected caption of **Figure 4** appears below.

“ELN grading (classified as NLSV versus LSV) on patient survival outcomes. In the intermediate-risk subgroup (A), no statistically significant difference in overall survival was observed between the NLSV and LSV groups (P = 0.437), while in the high-risk group (B), the LSV patients showed significantly lower overall survival rates than the NLSV patients (P = 0.043).

There were also mistakes in the abbreviation “ENL” throughout the article namely in section: **Introduction** and **Results**. All instances of “ENL” have been corrected to “ELN”.

Lastly, there was a mistake in the percentage notation, 28% was written as 280%.

A correction has been made to the section **Introduction**, Paragraph 1, 4^th^ sentence.

“A study examining overall survival (OS) rates in AML over the past 15 years [4] indicates that the 1-year, 3-year, and 5-year OS rates for patients diagnosed between 2011 and 2019 were 48%, 31%, and 28%, respectively, compared to 42%, 25%, and 22% for those diagnosed from 2004 to 2010.

The original version of this article has been updated.

